# Effect of Oral Contraceptive Use in Relation to Fertile Years on the Risk of Endometriosis in Women with Primary Infertility: A Ten-Year Single-Centre Retrospective Analysis

**DOI:** 10.3390/medicina60060959

**Published:** 2024-06-10

**Authors:** Vesna Šalamun, Gaetano Riemma, Anja Klemenc, Antonio Simone Laganà, Pasquale De Franciscis, Martin Štimpfel, Sara Korošec, Helena Ban Frangež

**Affiliations:** 1 Department of Human Reproduction, Division of Gynaecology and Obstetrics, University Medical Centre Ljubljana, 1000 Ljubljana, Slovenia; vesna.salamun@icloud.com (V.Š.); martin.stimpfel@kclj.si (M.Š.); sara_korosec@hotmail.com (S.K.); 2 Faculty of Medicine, University of Ljubljana, 1000 Ljubljana, Slovenia; klemenc.anja@gmail.com; 3 Obstetrics and Gynecology Unit, Department of Woman, Child and General and Specialized Surgery, University of Campania “Luigi Vanvitelli”, 80138 Naples, Italy; gaetano.riemma@unicampania.it (G.R.); pasquale.defranciscis@unicampania.it (P.D.F.); 4 Unit of Obstetrics and Gynecology, “Paolo Giaccone” Hospital, Department of Health Promotion, Mother and Child Care, Internal Medicine and Medical Specialties (PROMISE), University of Palermo, 90127 Palermo, Italy; antoniosimone.lagana@unipa.it

**Keywords:** endometriosis, oral contraceptives, staging, endometrioma, deep infiltrating endometriosis, infertility

## Abstract

*Background and Objectives:* Oral contraceptives (OCs) are usually used to treat endometriosis; however, the evidence is inconsistent about whether OC use in the past, when given to asymptomatic women, is protective against the development of future disease. We aimed to assess the relationship between the use of OCs and the likelihood of discovering endometriosis, considering the length of time under OCs during their fertile age. *Materials and Methods:* This was a monocentric retrospective cohort study in a tertiary-care University Hospital (Department of Human Reproduction, Division of Gynaecology and Obstetrics, University Medical Centre Ljubljana, Slovenia) carried out from January 2012 to December 2022. Reproductive-aged women scheduled for laparoscopic surgery for primary infertility and subsequent histopathological diagnosis of endometriosis were compared to women without an endometriosis diagnosis. They were classified based on the ratio of years of OC use to fertile years in four subgroups: never, <25%, between 25 and 50%, and >50. *Results:* In total, 1923 women (390 with and 1533 without endometriosis) were included. Previous OC use was higher in those with endometriosis than controls (72.31% vs. 58.64%; *p* = 0.001). Overall, previous OC usage was not related to histopathological diagnosis of endometriosis (aOR 1.06 [95% CI 0.87–1.29]). Women who used OCs for less than 25% of their fertile age had reduced risk of rASRM stage III endometriosis (aOR 0.50 [95% CI 0.26–0.95]; *p* = 0.036) or superficial implants (aOR 0.88 [95% CI 0.58–0.95]; *p* = 0.040). No significant results were retrieved for other rASRM stages. Using OCs for <25%, between 25 and 50%, or >50% of fertile age did not increase the risk of developing superficial endometriosis, endometriomas, or DIE. *Conclusions:* When OCs are used at least once, histological diagnoses of endometriosis are not increased. A protective effect of OCs when used for less than 25% of fertile age on superficial implants may be present. Prospective research is needed to corroborate the findings due to constraints related to the study’s limitations.

## 1. Introduction

Between 5% and 10% of women who are of reproductive age have endometriosis, a major health issue that is described histologically as functioning endometrial glands and stroma growing outside of the uterine cavity [1]. Female pelvic pain and infertility resulting from damage to the peritoneal tissues are frequently caused by these ectopic uterine implants, which are associated with inflammation mediated by sex hormones [2]. Due to the substantial financial burden that endometriosis causes, it has emerged as a major public health concern [3].

An ongoing history of dysmenorrhea, which is frequently primary and severe, is a typical early clinical sign of endometriosis [4]. When non-steroidal anti-inflammatory medicines (NSAIDs) fail to provide enough relief from dysmenorrhea to young women, oral contraceptives (OCs) are frequently recommended as a therapy option [5]. The symptoms of dysmenorrhea are alleviated while ovarian function is suppressed by OCs, but they typically return after cessation. Practically speaking, because OCs have been demonstrated to successfully prevent symptom recurrence, they are frequently continued for several years [5]. 

Moreover, given the high frequency of OC use, particularly among young women who require contraception, and the concerningly high prevalence of endometriosis in this population, any association between the two factors—either one that increases or decreases risk—would have far-reaching implications [6]. Furthermore, because OCs are used to treat symptomatic endometriosis, it would be crucial to understand any possible consequences this kind of medication may have on the course of the illness.

Oral contraceptives (OCs) are commonly used to treat endometriotic patients; however, the evidence is inconsistent about whether OC use in the past, when given to healthy, asymptomatic women, is protective against the development of endometriosis in the future [7]. The idea that earlier exposure to exogenous hormones may lessen the total quantity of endometrial tissue exposed to the peritoneal cavity through retrograde menstruation supports the possibility of protection [8]. In addition, it has been demonstrated that OC exposure prevents the development of endometriosis in a chicken model of this gynecologic disorder [9]. In contrast, a meta-analysis of endometriosis has demonstrated that there may be a higher risk, although non-significant, of endometriosis associated with past exposure to OCs [10]. Subsequent research has found a similar pattern [7]. Later, another study showed that past but not current exposure to OCs is associated with endometriosis [11].

Some previous reports hypothesized a theoretical role of OCs in endometriosis pathogenesis. However, the mechanism underlying a plausible impact was considered intricate. Significant influences may come from environmental variables as well as genetic predisposition. The usage of OCs may affect a woman’s genetic vulnerability to endometriosis. Some women may be more prone to developing endometriosis than others [10]. Menstrual flow patterns can be changed by oral contraceptives. Retrograde menstruation, or the backward flow of menstrual blood into the pelvic canal, is one theory for how endometriosis develops, and, in certain situations, this may make it more likely [8,9]. According to another hypothesis, endometrial cells found in menstrual blood could proliferate and implant outside of the uterus. Some authors have reported that the hormonal milieu that OCs produce may cause alterations in endometrial cell activity. These modifications may lead to the development of endometriosis by increasing the adherence, invasiveness, and survival of endometrial cells beyond the uterine cavity [7,8,9]. Conversely, such theories were abandoned as most available reports show that the risk of endometriosis under OC therapy appears reduced, increasing the evidence supporting a protective effect of OCs on the beginning and progression of the disease [10].

Because it is still unclear how endometriosis and present or past OC usage are related, we decided to reevaluate the issue by taking into consideration the two most likely sources of ambiguity: the fraction of OC users who use them chronically and the location and extension of endometriosis. 

Therefore, our study aimed to evaluate how, considering the amount of time spent under OC therapy in their fertile age, the use of OCs should be related to the risk of finding endometriosis at surgical and subsequent histopathological diagnosis.

## 2. Materials and Methods

The present investigation was structured as a retrospective examination of data that were prospectively gathered from women who underwent primary infertility surgery at the Department of Human Reproduction, University Medical Centre of Ljubljana, between 1 January 2012 and 31 December 2022.

The design, analysis, interpretation, drafting, and revision stages have all taken into account the Helsinki Declaration, the Committee on Publication Ethics (COPE) guidelines (http://publicationethics.org/, accessed on 31 March 2024), and the Reporting of studies Conducted using Observational Routinely-collected Health Data (RECORD) Statement of the STrengthening the Reporting of OBservational studies in Epidemiology (STROBE) collaborative, made available through the enhancing the quality and transparency of health research (EQUATOR) network (https://www.equator-network.org, accessed on 31 March 2024). Any information that could be used to formally identify the patient was removed from the data through anonymization. Every participant in this study signed a consent form granting permission for data collection and analysis for research purposes, and they were informed about the procedures. The study was approved by the Medical Ethics Committee of the Republic of Slovenia with approval ID no. 0120-536/2023-2711-5.

The inclusion criteria for the study considered women of reproductive age, from 18 to 45 years old, diagnosed with primary infertility, defined, according to World Health Organization criteria, as a couple who, despite trying for at least a year through unprotected sexual activity, has never been able to conceive, and who were scheduled for planned laparoscopic surgery for infertility cause assessment. After surgery, women were classified as patients with or without endometriosis. In the case of endometriotic patients, a confirmed histopathological diagnosis of endometriosis upon surgical removal of the lesion was needed. Women were considered non-endometriotic when no endometriosis was found upon laparoscopy or it was not confirmed upon subsequent histopathological analysis.

In cases where a woman was symptomatic (complaining of chronic pelvic pain, dysmenorrhea, or dyspareunia), deemed unsuitable for or declined a laparoscopic approach, had a preoperative diagnosis of a malignant disease, either gynecological or non-gynecological, or had severe systemic illnesses (such as autoimmune or endocrine diseases, severe coagulopathy, or cardiac pathology), she was excluded from the study. Additionally, individuals who tested positive for premalignancy or malignancy during a postsurgical histological examination were excluded from the analysis. 

Subsequently, women were additionally subdivided according to the ratio of years of OC use to fertile years, expressed as a percentage, defined as years from menarche to surgery, into four subgroups: 0 (never used), less than 25%, between 25% and 50%, and more than 50% of fertile years.

We also gathered basic data for each patient, including age, BMI, menarche age, length of menstrual cycle, duration of infertility, previous use of OCs, and cumulative number of years on OCs. Endometriotic lesions were categorized into three classes based on histological findings: endometrioma/s, deep infiltrating endometriosis (DIE), and superficial (peritoneal). When endometriotic lesions disrupted the muscularis of pelvic organs, DIE was histologically defined. Endometriotic patients were categorized as having the most prominent finding because these three forms of endometriotic lesions are often related to one another. DIE, endometrioma(s), and superficial endometriotic lesions were ranked from most to least bothersome. For instance, a patient was labeled as DIE when they presented with superficial lesions linked to DIE nodules. Endometriosis was also staged according to the revised American Society for Reproductive Medicine (rASRM) criteria [12]. 

### Statistical Analysis

First, the distribution of continuous variables was checked for normality. They were then reported as mean ± standard deviation (SD), and the independent samples’ *t* test was used to assess group differences. The chi-square test was used to compare the groups based on categorical data, which were expressed as numbers (percentages).

The possible risk factors for histological endometriosis diagnosis at surgery were screened and determined through univariate and multivariate logistic regression analysis.

The relationship between the percentage of use of OCs according to fertile age and the rASRM score or presence of superficial, DIE, or endometrioma/s was evaluated using univariate regression analysis. A *p*-value (*p*) < 0.05 was considered statistically significant. STATA 14.1 (StataCorp. LLC, College Station, TX, USA) software was used for all of the analyses of retrieved data.

## 3. Results

In total, 1923 infertile women were deemed suitable for the analysis after the exclusion of 138 women based on the abovementioned criteria. Of those, 390 (20.28%) patients had histologically proven endometriosis, and 1533 (79.72%) were controls with no visualized or histopathologically confirmed lesions.

Main characteristics of the two cohorts are reported in Table 1. 

The mean number of years under OC treatment was higher in endometriotic rather than non-endometriotic patients (4.22 ± 4.29 vs. 2.97 ± 3.79; *p* < 0.001). Considering the mean duration of OC treatment among the subtypes of endometriosis (superficial, endometriomas, and DIE), there were no significant differences (4.23 ± 4.08 vs. 4.09 ± 4.15 vs. 4.36 ± 4.82 years) for superficial, endometriomas, or DIE, respectively (*p* = 0.216). 

Similarly, previous OC use was significantly higher in the endometriosis group relative to controls (72.16% vs. 62.10%; *p* = 0.001). When stratified using the number of years on OC therapy relative to years of fertile age, significant differences between the two groups were noted (Table 1; *p* = 0.001). 

Subsequently, women with superficial endometriosis, endometriomas, and DIE were stratified according to the percentage of OC usage relative to years of fertile age, showing significant differences between no usage, less than 25%, between 25 and 50%, and more than 50% compared to women with no endometriosis (Table 2).

Overall, there was no increased chance of being diagnosed with endometriosis in infertile women who used OCs at least once in their fertile age (aOR 1.06 [95% CI 0.87–1.29]).

The relationship with surgical staging according to rASRM classification is reported in Table 3. Women who used OCs for less than 25% of their fertile age had a 50% reduced (aOR 0.50 [95% CI 0.26–0.95]; *p* = 0.036) risk of developing rASRM stage III endometriosis. No other significant results were notable for rASRM I, II, or IV stages (Table 3).

Similarly, the use of OC for less than 25% of fertile age was related to a 12% reduction in the risk of developing superficial endometriosis (aOR 0.88 [95% CI 0.58–0.95]; *p* = 0.040) Conversely, the diagnosis of endometriomas and DIE seemed to be unaffected by the percentage of OC usage across the years (Table 3).

## 4. Discussion

This study showed that the use of OCs seems to avoid an increase in the surgical and histopathological diagnosis of endometriosis in infertile women. A protective effect of the use of OCs for less than 25% of fertile years could be retrievable for moderate and superficial endometriosis.

There are several speculations related to these findings. First and foremost, understanding endometriosis medical therapy depends in large part on the hormone concentrations found in the peritoneal fluid. Reductions in estrogen concentrations rather than a direct progestin action appear to be the reason behind the effects of oestro-progestin therapy on superficial endometriosis lesions [13,14,15].

Nonetheless, progesterone concentrations in the peritoneal fluid are incompatible with a histologically proliferative aspect that lacks secretory alterations in most superficial and mild lesions [16]. Thus, a significant progesterone resistance in these lesions must be hypothesized. The exact mechanism is unknown, but it may be a peritoneal fluid effect in women with endometrial defects that predispose them to progesterone, or it could be isolated endometrial glands with progesterone resistance, or it could be subtle lesions originating from the basal endometrium [17]. The latter possibility is appealing because progesterone does not cause secretory changes in the basal endometrium, but withdrawal of progesterone—which does not occur in peritoneal fluid—is necessary to resume mitotic activity and proliferation [18,19]. 

Consequently, given the inherent properties of superficial implants and their crosstalk with the peritoneal microenvironment, we can also speculate that endocrine system malfunction plays a part, impacting local macrophage function via overexpression of estrogen and progesterone resistance [20,21]. An aberrant immunological milieu is produced by overexpressed estrogen and the estrogen receptor (ER) on peritoneal cavity macrophages. Aromatase and 17b-hydroxysteroid dehydrogenase catalyze the increased synthesis of estrogen and decreased metabolism of 17b-estradiol [22,23]. Supplementing with OCs may, for the first years of use, overcome such a mechanism and reduce the incidence of de novo peritoneal implants, while, on long-term therapy, such an effect might be mitigated due to the changes in the crosstalk between superficial implants and the peritoneal microenvironment.

When used for more or less than 50%, the usage does not seem to increase the risk of developing the disease. Moreover, OC usage does not seem to be related to increased or reduced DIE and endometrioma diagnoses.

According to this finding, we could speculate that there is a long-term positive effect of adherence to OC therapy, with the progestogen-mediated antiproliferative effects overcoming the estrogen-related proliferative characteristics of superficial endometriotic implants [24]. Meanwhile, the adherence to therapy and the increased usage of OCs does not seem to interfere with either DIE or endometriomas.

**Figure 1:**
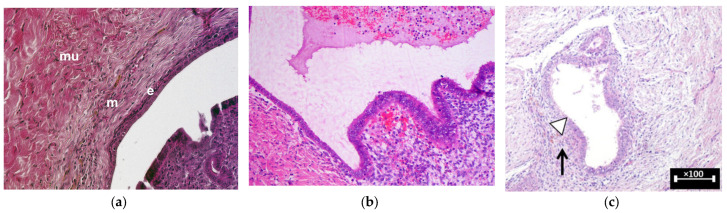
Histopathologic pictures (hematoxylin/eosin staining) of different morphological aspects of endometriosis localizations. (**a**) Superficial endometriosis; ‘mu’ = muscle; ‘m’ = myometrium; ‘e’ = endometrial tissue. Adapted from Schreinemacher MH, et al. *PLoS One*. 2012 [25]. (**b**) Ovarian endometrioma. Adapted from Gałczyński, et al. *J Ovarian Res*. 2019 [26]. (**c**) DIE; endometrial glands (white arrowhead) in the peritoneal structure with stromal and inflammatory infiltrate (black arrow) in the sub-mesothelial connective tissue. Adapted from Istrate-Ofiţeru, A.-M., et al. *IJMS*, 2024 [27].

Moreover, the influence of OCs on peritoneal lesions is more pronounced as these lesions are reported to be more histopathologically similar to eutopic endometrium compared to endometriomas and DIE, making them different histopathological entities (Figure 1) [25,26,27].

Therefore, OCs may not have play a prophylactical role in the case of endometriomas and DIE due to a different pathophysiological mechanism that makes the less prone to OC action [28].

When it comes to the potential for primary prevention, Missmer et al. [29] asserted that the prescription of OC before endometriosis onset should be a valid health intervention, citing the finding that the ovulatory-cycle-associated risk of endometriosis appears to be highest among those who have never used OCs [26]. However, a significant need for large-scale trials is necessary before recommending OCs for primary disease prevention. In fact, even if we had enough evidence to exclude the association between past OC use and endometriosis, the evidence still remains of low quality. In fact, to date, only retrospective analyses and quantitative syntheses have analyzed this issue. Prospective studies in the future should ascertain whether OC use is an ascertained protective factor not only for peritoneal endometriosis or if it plays a pivotal role in DIE. Also, until adolescent women desire to become pregnant, OCs would be prescribed to them on a systematic basis. This would result in significant organizational issues, the need to spend money on health care resources, and the possibility of both organic and psychological morbidity [10].

Moreover, approximately one-third of women using hormonal contraception treatment are said to not respond to treatment. Women who are resistant to treatment for progesterone may be suffering from an imbalance of adhesion molecules or estrogen and progesterone receptor subtypes. Dynamic monitoring of the therapeutic response is necessary because there are no biomarkers that predict progesterone resistance. This allows for the possibility of considering surgical treatment or changing to an alternative medical course of treatment [30]. In our study, we were not able to assess the difference between women who stopped treatment early because of unresponsiveness or because they experienced certain adverse effects connected to hormone metabolism. Expert opinions have recently recommended the cautious use of more costly, but more successful, medications in women who are intolerant of, do not respond to, or are otherwise contraindicated for hormonal contraceptives. It was recently suggested to use a stepped-care strategy that would use OCs as the first step, progestins (including progestin-only contraception) as the second step, and Gonadotropin-Releasing Hormone agonists and antagonists as the third step. This strategy may lessen the cost of medical care for both patients and healthcare providers [31]

However, this protective association between OCs and endometriosis does not simply imply that a limited use of OCs avoids the risk of endometriosis. While interpreting data, potential confounding factors and limitations need to be considered.

The findings are limited by inherent limitations of regression model analyses, as there could be unstudied variables that might influence and interfere with the main outcomes. Although we tried to avoid such issues by reducing the number of independent variables and standardizing the surgical approach, overfitting may not be excluded. However, as previously mentioned, to reduce disparities between endometriosis patients and non-endometriotic controls, we also selected solely infertile patients to avoid biased estimates produced from regression analysis, and we have a single major inclusion criterion. Compared to the previous studies on the topic, this is a strength of our research.

Because oral contraception is commonly administered as first-line treatment for dysmenorrhea, a symptom strongly associated with endometriosis, selection biases may have occurred. To avoid the possibility of bringing on too many patients who need surgery after their medical treatments’ failure, we excluded women with dysmenorrhea or dyspareunia and chronic pelvic pain, focusing only on primary infertility patients. In fact, the number of endometriosis cases determined by eliminating symptom presentation in any population will always be underestimated due to the use of medications, including OCs, for contraception, as up to 44% of surgically diagnosed cases of endometriosis that are left untreated will spontaneously resolve at follow-up surgery, highlighting the syndrome’s intrinsic variability [11,32,33]. 

Similarly, this is the first study to evaluate how the quantity of OC use (and not only the possibility of past or current OC use) might have an impact on the possibility of diagnosing endometriosis during surgery.

## 5. Conclusions

Infertile women’s surgical and histological diagnoses of endometriosis appear not to be higher when OCs are used. When a woman uses OCs for less than 25% of her reproductive years, there could be a protective effect on avoiding peritoneal implants and moderate-stage pathology. However, due to limitations accountable to the retrospective design of the study, further prospective research is required to validate the available findings. 

## Figures and Tables

**Table 1:** Main characteristics of evaluated cohorts.

	Endometriosis	N = 390	Controls	N = 1533	*p*-Value
	Mean	SD	Mean	SD	
Age, years	32.20	4.46	32.37	4.86	0.531
BMI, kg/m^2^	24.07	3.66	24.48	6.90	0.318
Length of the cycle, days	27.98	3.23	28.75	4.71	0.115
Days of bleeding	5.16	1.43	5.18	1.41	0.803
Menarche, years	12.98	1.46	12.92	1.68	0.518
Fertile years, years	19.22	4.67	19.46	5.01	0.392
Years on OC	4.29	4.41	2.84	3.74	<0.001
	N	%			
Previous OC use	282	72.31%	899	58.64%	0.001
% OC use/fertile years					
0	108	27.69%	634	41.36%	0.001
<25%	118	30.26%	476	31.05%	
25–50%	110	28.21%	312	20.35%	
>50%	54	13.85%	111	7.24%	
rASRM Classification					
I	205	52.56%			
II	77	19.74%			
III	83	21.28%			
IV	19	4.87%			
Superficial Endometriosis	139	35.64%			
Endometrioma/s	142	36.41%			
DIE	109	27.95%			

SD: standard deviation; OC: oral contraceptive.

**Table 2:** Women with superficial endometriosis, endometrioma/s, and DIE according to OC use.

	Superficial		Endometrioma/s		DIE		Controls		*p*-Value
% OC Use/Fertile Years	N	%	N	%	N	%	N	%	
0	48	34.53%	37	26.06%	23	21.10%	634	41.36%	0.001
<25%	52	37.41%	27	19.01%	39	35.78%	476	31.05%	
Between 25 and 50%	27	19.42%	55	38.73%	28	25.69%	312	20.35%	
>50%	15	10.79%	20	14.08%	19	17.43%	111	7.24%	

**Table 3:** Univariate analysis for endometriosis staging and location according to OC use.

		aOR	95% CI	*p*-Value	aOR	95% CI	*p*-Value	aOR	95% CI	*p*-Value
	0	<25%			Between 25 and 50%			>50%		
rASRM I	Ref.	0.83	0.49–1.40	0.479	0.86	0.51–1.47	0.580	0.87	0.45–1.66	0.656
rASRM II	Ref.	0.77	0.40–1.77	0.381	1.05	0.74–2.14	0.636	1.10	0.38–3.15	0.856
rASRM III	Ref.	0.50	0.26–0.95	0.036	0.69	0.37–1.28	0.238	0.66	0.30–1.45	0.308
rASRM IV	Ref.	0.54	0.13–2.30	0.403	0.78	0.20–2.29	0.713	3.06	0.82–6.60	0.101
Superficial Endometriosis	Ref.	0.88	0.58–0.95	0.040	1.25	0.73–2.13	0.412	1.07	0.55–2.09	0.823
Endometrioma/s	Ref.	0.98	0.57–1.70	0.959	1.21	0.69–2.12	0.504	0.82	0.42–1.60	0.567
DIE	Ref.	1.82	0.98–3.32	0.070	1.26	0.67–2.37	0.469	2.00	0.97–4.13	0.059

aOR: Adjusted Odds Ratio; Ref.: Reference; CI: Confidence Interval. aOR adjusted for age, BMI, and fertile years.

## Data Availability

The original contributions presented in the study are included in the article; further inquiries can be directed to the corresponding author.
